# External hinged fixation vs. internal joint stabilization for elbow instability: a systematic review and meta-analysis of functional outcomes and surgical complications

**DOI:** 10.1016/j.xrrt.2025.100638

**Published:** 2025-12-11

**Authors:** Areeb Ahmad, Kassem Ghayyad, Alaina Mitchell, Daryl C. Osbahr, G. Russell Huffman, Luke S. Oh, Amir R. Kachooei, Rothman Florida Elbow Experts (ROFLEX) Group

**Affiliations:** a Rothman Orthopaedics Florida at AdventHealth, Orlando, FL, USA; b University of Central Florida College of Medicine, Orlando, FL, USA; c New York Institute of Technology College of Osteopathic Medicine, Westbury, NY, USA; d Rothman Florida Elbow Experts (ROFLEX) Group, Orlando, FL, USA

**Keywords:** Elbow instability, Hinged external fixator, Internal joint stabilizer, Disabilities of the arm, Shoulder and hand, Nerve injury, Range of motion, Systematic review

## Abstract

**Background:**

Traumatic elbow instability can be managed with hinged external fixator (HEF) or internal joint stabilizer (IJS). While prior studies report device-related complications with both devices, a comprehensive analysis comparing range of motion (ROM), patient-reported outcome measures, and surgical complications is limited. This study aims to evaluate these outcomes to guide treatment decisions for complex elbow instability.

**Methods:**

This systematic review and meta-analysis was conducted following Preferred Reporting Items for Systematic Reviews and Meta-Analyses guidelines. A comprehensive search was performed in Google Scholar and PubMed from January 1, 2000, to February 20, 2025. Level I-IV studies were included if they reported on postoperative ROM, patient-reported outcome measure, or surgical complications as outcome measures in patients treated with HEF or IJS for elbow instability.

**Results:**

Of the 2,041 articles identified, 38 studies met inclusion criteria for quantitative synthesis, including 27 of moderate quality and 11 of high quality based on the Newcastle–Ottawa Scale classification. Across the 29 retrospective studies, 8 prospective studies, and 1 randomized control trial, 500 patients underwent treatment with HEF, while 263 patients were treated with IJS. Disabilities of the Arm, Shoulder, and Hand scores were significantly better in the HEF group compared to IJS (9.8 vs. 23; *P* < .001). No significant differences were found between HEF and IJS in postoperative ROM, Mayo Elbow Performance Index, visual analog scale for pain, heterotopic ossification, or nerve injury rates.

**Conclusion:**

HEF and IJS showed comparable rates of postoperative ROM, Mayo Elbow Performance Index, visual analog scale, heterotopic ossification, and nerve injury. However, Disabilities of the Arm, Shoulder, and Hand scores were 13.2 points lower in the HEF group, exceeding the minimal clinically important difference of 10.8 and indicating a clinically meaningful functional advantage of the upper extremity. This difference may be influenced by the less invasive nature of hardware removal with HEF compared to IJS. These findings should be interpreted with caution, given the overall lower level of evidence and heterogeneity across studies. Future prospective investigations with standardized rehabilitation protocols, longer follow-up, and stratification by injury chronicity, limb dominance, and preoperative motion are needed to better define optimal indications for each technique.

Following the repair and reconstruction of an elbow fracture dislocation, or after a comprehensive joint release that results in instability, a temporary fixation device is applied to secure the repair and preserve the congruity of the elbow joint until the ligaments have healed. This additional fixation can include intra-articular pins, bridge plates, or static external fixators, which typically stabilize the elbow at a 90-degree angle.^28^ In contrast, hinged external fixators (HEF) and internal joint stabilizers (IJS) facilitate an immediate, controlled range of motion (ROM) while maintaining joint congruity.^1^

HEF and IJS have gained prominence as temporary fixation methods that enable stability, early mobilization, and satisfactory outcomes in patients with recurrent or persistent instability following surgical reconstruction or post-traumatic complex instability.^18^^,^^38^^,^^51^ Multiple studies have reported improvements in both postoperative ROM and patient-reported outcome measures (PROMs) using HEF and IJS.^13^^,^^48^ A recent systematic review described device-related complications between the 2 methods, noting higher, but not statistically significant, rates of pin-site infections, construct failure, and recurrent instability with HEF.^14^ However, a comprehensive analysis directly comparing functional outcomes and surgical complications between HEF and IJS is limited. This systematic review and meta-analysis aim to compare postoperative PROM, ROM, and surgical complication profiles associated with HEF and IJS in the management of elbow instability. The primary null hypothesis was that there is no difference in postoperative functional outcomes between patients treated with HEF and those treated with IJS. The secondary null hypothesis was that there is no difference in surgical complication rates between the 2 techniques.

## Methods

### Search strategy

This systematic review adhered to the Preferred Reporting Items for Systematic Reviews and Meta-Analyses guidelines.^21^ Two authors AA and KG conducted a systematic search using Google Scholar and PubMed from January 1, 2000, to February 20, 2025. The search strategy utilized the keywords and controlled vocabulary detailed in Supplementary Figure S1.

### Eligibility criteria

Two authors AA and KG independently screened titles and abstracts. Duplicates, non-English papers, case reports, and biomechanical/cadaveric studies were excluded. The full texts of the potentially eligible articles were then screened, considering the inclusion and exclusion criteria. The inclusion criteria were: (1) human studies including patients with acute or chronic post-traumatic elbow instability; (2) studies in which patients were treated with either HEF or IJS; (3) comparative and single-arm studies in which results for HEF and IJS could be stratified; (4) studies reporting at least one of the following outcomes: surgical complications, PROM and preoperative, and/or postoperative ROM. Exclusion criteria included: (1) studies focusing on distal humerus fractures, as this review evaluates instability resulting from elbow dislocations, ligamentous injuries, and fractures of the coronoid process or radial head; (2) studies where patients underwent HEF or IJS for conditions unrelated to elbow instability; (3) studies including pediatric patients <18 years; (4) studies with a sample size of fewer than 5 patients; (5) studies in which outcomes for HEF and IJS could not be separately analyzed; (6) studies that did not report on surgical complications, PROM, or ROM; and (7) patients managed with static external fixators, bridge plates, or other stabilization methods were excluded, as these modalities were outside the scope of this review.

### Selection process and data extraction

Following the screening of titles and abstracts based on the preliminary inclusion criteria, full-text articles were retrieved and screened by both authors AA and KG. Any discrepancies during this phase were resolved through discussion with the senior author ARK. Two reviewers, AA and AM, separately collected data from the full texts of the included studies using a predesigned Excel spreadsheet (Microsoft Excel; Microsoft Corp., Redmond, WA, USA). The extracted data were compared and cross-checked, with discrepancies resolved through consensus and discussion with the senior author ARK.

The extracted data included demographic variables (total sample size, mean age, age range, sex distribution, body mass index, laterality, and classification of acute vs. chronic instability), surgical details (injury type, surgical device, and time from instability to surgery for acute and chronic cases), and follow-up duration (mean, median, and range). Preoperative and postoperative ROM data were recorded, including flexion–extension arc, flexion contracture, pronation, supination, and total forearm rotation for both acute and chronic instability. PROM included the visual analog scale (VAS) for pain, Disabilities of the Arm, Shoulder, and Hand (DASH) score, and Mayo Elbow Performance Index (MEPI). Complications were assessed based on the reported incidence of heterotopic ossification (HO) and nerve injury, with data extracted separately for acute and chronic instability when available.

For PROM and ROM, the reported standard deviation (SD) was extracted when available. If SD was not provided but the study included mean, range, and individual patient data, SD was manually calculated. If only mean and range were reported without individual patient data, SD was not calculated, and the data were excluded from the analysis.

### Statistical analysis

After data extraction, each study was reviewed to determine eligibility for meta-analysis. To be included, at least one outcome required data from a minimum of 2 studies for both HEF and IJS. Most studies were single-arm, except for one comparative clinical trial study, in which data for HEF and IJS were analyzed separately.^54^

To compare rates of surgical complications, a proportional meta-analysis was performed using R Studio, followed by a subgroup comparison using chi-square (χ^2^) and degrees of freedom (df). A *P* value < .05 was considered statistically significant. For postoperative PROM and ROM, a meta-mean analysis was conducted in R Studio, with subgroup differences assessed using χ^2^ and df. A *P* value < .05 was considered statistically significant.

Since this is a systematic review and meta-analysis synthesizing previously published data, a traditional a priori power analysis was not applicable. Instead, we assessed the adequacy and precision of the pooled estimates through confidence intervals (CIs), heterogeneity testing (I^2^), and evaluation of publication bias. Heterogeneity was evaluated using the *I*^*2*^ statistic, with values > 50% indicating substantial heterogeneity. A fixed-effects model was applied when *I*^*2*^ ≤ 50%, whereas a random-effects model was used when I^2^ > 50%. Intervention arms with fewer than 5 patients were excluded to minimize bias from small sample sizes. Preoperative ROM and PROM data were insufficient to conduct a meaningful meta-analysis. In addition, publication bias was evaluated by visual inspection of funnel plots for asymmetry across all analyzed outcome measures.

### Risk of bias

We assessed the quality of the included studies using the Cochrane Risk of Bias Assessment Tool (RoB 2) for the randomized controlled trial (RCT) and the Newcastle–Ottawa Scale (NOS) for the retrospective and prospective cohort studies.

The RoB 2 evaluates 5 key domains: randomization process, deviations from intended interventions, missing outcome data, measurement of the outcome, and selection of the reported result. Studies are categorized as having a low, some concerns, or high risk of bias for each domain, with an overall risk of bias rating assigned.^49^

The NOS evaluates observational study quality across 3 domains: (1) selection (up to 4 points), which considers how representative the exposed and nonexposed cohorts are, how exposure was determined, and whether the outcome was absent at baseline; (2) comparability (up to 2 points), which assesses how well the study accounts for potential confounding variables; and (3) outcome (up to 3 points), which evaluates outcome assessment methods, adequacy of follow-up duration, and completeness of follow-up.^47^ Studies scoring ≥ 7 points are generally considered to have higher methodological quality, while those scoring 4-6 points are considered moderate quality, and those scoring ≤ 3 points indicate a higher risk of bias and lower methodological rigor.

## Results

### Study selection

**Figure 1:**
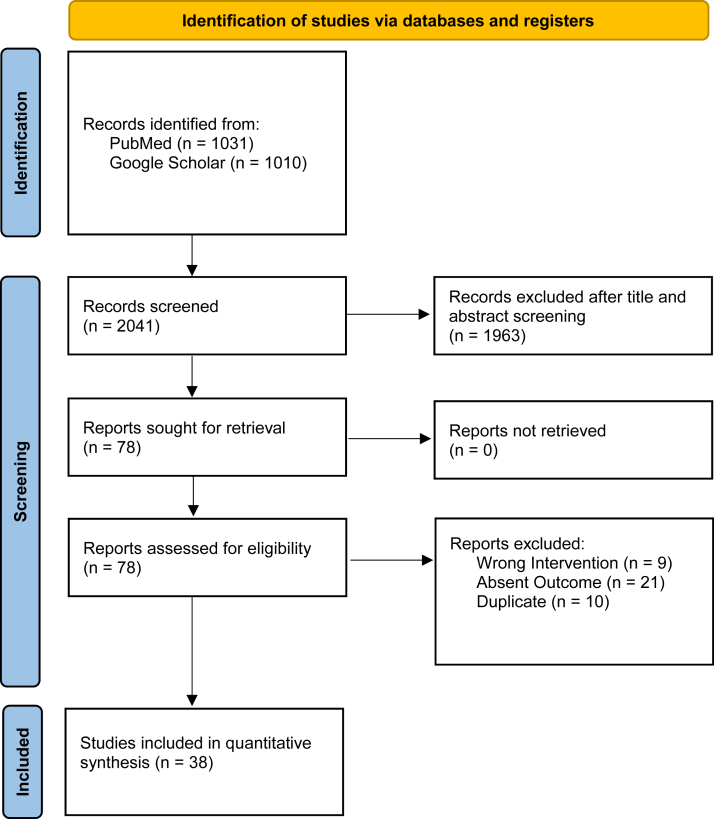
PRISMA diagram. *PRISMA*, Preferred Reporting Items for Systematic Reviews and Meta-Analyses.

A total of 2,041 articles were retrieved across PubMed and Google Scholar, 78 of which met the preliminary inclusion criteria after title and abstract screening. After the final stage of screening, 38 studies remained for our quantitative synthesis (Fig. 1).

### Bias and quality assessment

Thirty-eight cohort studies were assessed using the NOS. Of these, 11 studies (29%) were classified as high quality (≥ 7 points), while 27 studies (71%) were categorized as moderate quality (4-6 points) (Supplementary Table S1). The single RCT included in this analysis was evaluated using RoB 2, which indicated an overall low risk of bias (Supplementary Table S2). Most moderate studies had limitations in confounder adjustment and follow-up consistency; however, the evidence provides a reasonable basis for the analysis of functional outcomes and surgical complications following HEF and IJS.

### Study characteristics

All 38 studies included in this analysis were published between 2000 and 2025. Of these, 29 were retrospective studies, 8 were prospective studies, and 1 was a RCT. One of the retrospective studies was a comparative, double-arm study that included both HEF and IJS; data from each intervention group were separately extracted and analyzed.^54^ Across all studies, a total of 500 patients underwent treatment with HEF, while 263 patients were treated with IJS. The included studies were comparable in mean patient age and sex distribution (Table I).

**Table I:** Study characteristics.

First author, yr	Study type	Sample size (n)	Age (mean)	Male (%)	Dominant arm injured (%)	Surgical device	Mean time from injury to surgery	Mean follow-up	Follow-up range
Ruch, 2001^40^	Retrospective	8	55	88	38	HEF		189 d	111-302 d
Von Knoch, 2001^51^	Retrospective	11	40	73		HEF		35 weeks	9-133 weeks
Jupiter, 2002^18^	Prospective	5	49	40	20	HEF	11 weeks	38 mo	12-98 mo
Stavlas, 2003^47^	Prospective	8	63	62		HEF		11 mo	8-15 mo
Ring, 2004^39^	Retrospective	13	45	69	77	HEF	2 mo	57 mo	24-81 mo
Papandrea, 2007^32^	Retrospective	16	44	69		HEF	11 weeks	>5 yr	9 months-13 yr
Yu, 2007^54^	Retrospective	20	43	75	65	HEF	26 d	2.1 y	1-7.1 y
Lindenhovius, 2008^21^	Retrospective	13	48	77	92	HEF	7 weeks	34 mo	9-66 mo
Zilkens, 2009^55^	Retrospective	24	55	50		HEF	88 d	10.6 mo	2.9-27.7 mo
Sorenson, 2011^49^	Retrospective	17	44	65		HEF	11.2 weeks	44 mo	12-83 mo
Maniscalco, 2014^26^	Prospective	19	74.5	32		HEF		12 mo	
Ring, 2014^38^	Retrospective	19	47	63		HEF		31 mo	5-83 mo
Wang, 2014^52^	Retrospective	46	37	54		HEF	11 mo	24.3 mo	18-33 mo
Hopf, 2015^14^	Retrospective	26	53.3	54		HEF		51.7 mo	6-107 mo
Iordens, 2015^16^	Prospective	26	48	46	50	HEF			
Potini, 2015^36^	Retrospective	7	37	29	43	HEF	8 mo	41 mo	16-106 mo
Castelli, 2016^4^	Retrospective	11	41	73		HEF	3 d	5 weeks	1-6 mo
Pizzolo, 2016^35^	Retrospective	32	64	41	47	HEF	8.2 d	47 mo	12-95 mo
Sakai, 2016^41^	Retrospective	11	57.6	45		HEF	13.5 d	15.7 mo	10-26 mo
Orbay 2017^31^	Prospective	24	57	50		IJS		10 mo	1 d-26 weeks
Sochol, 2018^44^	Retrospective	20	48.8	65		IJS		16.3 mo	2-25 mo
Pasternack, 2020^34^	Retrospective	10	50.8	40	40	IJS		401 d	118 to 836 d
AlQahtani, 2021^2^	Retrospective	24	56	67		HEF		25 mo	13-36 mo
Chamseddine, 2021^5^	Retrospective	13	42	69		HEF	1 mo	7 yr	12-18 y
Pardo-Garcia, 2021^33^	Retrospective	5	37.4	80		IJS	3 d	9.8 mo	6-12 mo
Fene, 2022^11^	Retrospective	17	41	53		IJS	13 d	9 mo	2.5-24 mo
Ma, 2022^24^	Retrospective	17	48	71		HEF			12-24 mo
Meccariello, 2022^29^	Retrospective	46	43	70	46	HEF		28 mo	12-36 mo
Salazar, 2022^42^	Retrospective	22	46	55		IJS		12.5 mo	3-26.5 mo
Sheth, 2022^43^	Retrospective	30	43	77	43	IJS		10 mo	
London, 2024^22^	Retrospective	29	50.9			IJS			3.2-9.5 mo
Lu, 2023^23^	RCT	21	39.86	52	86	HEF	4.19 mo	31.95 mo	
Uddin, 2023^46^	Retrospective	12				IJS			2-24 weeks
Wynn, 2023^53^	Retrospective	12	52 (IQR)	50		IJS	17 d		
Wynn, 2023^53^	Retrospective	12	58 (IQR)	42		HEF	14 d		
De Crescenzo, 2024^9^	Prospective	16	45.1	69	69	IJS	4.58 mo		12-22 mo
De Crescenzo, 2024^10^	Prospective	10	42.6	40		IJS		13.5 mo	11-17.5
Heifner, 2024^13^	Retrospective	56				IJS		41 mo	
Ma, 2024^25^	Prospective	25	33.76	76	56	HEF			6-48 weeks

*IJS*, internal joint stabilizer; *RCT*, randomized controlled trial; *IQR*, interquartile range.

### Disabilities of the arm, shoulder, and hand

A total of 3 studies, including 71 patients treated with HEF,^15^^,^^36^^,^^40^ and 9 studies, including 168 patients treated with IJS,^9^, ^10^, ^11^^,^^23^^,^^32^^,^^35^^,^^44^^,^^45^^,^^50^ reported DASH scores. Heterogeneity analysis for the HEF group yielded *I*^*2*^ = 55%, indicating moderate variability among studies. A random-effects model was applied, with a pooled mean postoperative DASH score of 9.8 (95% CI: 5.4-14). Conversely, heterogeneity analysis for the IJS group demonstrated *I*^*2*^ = 98%, indicating substantial variability, prompting the use of a random-effects model, with a pooled mean postoperative DASH score of 23 (95% CI: 17-29).

**Figure 2:**
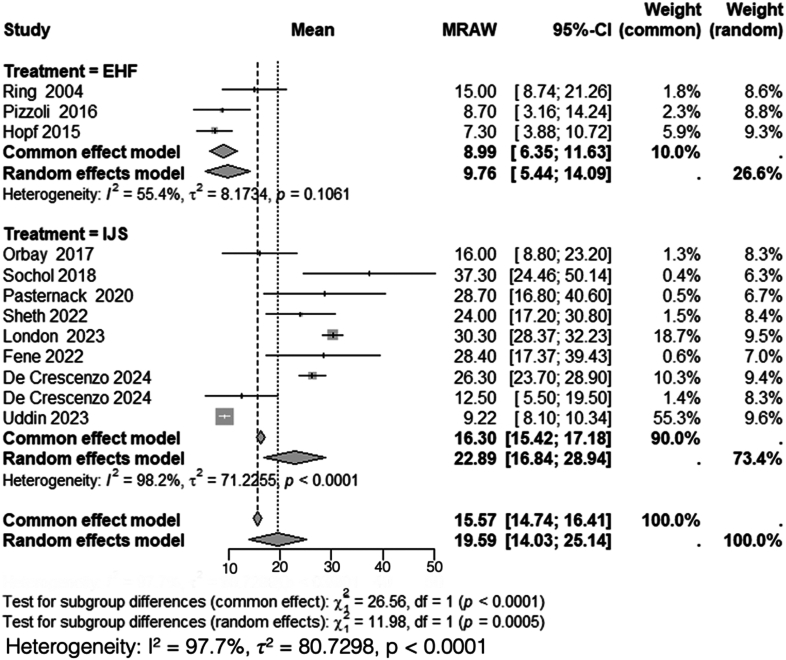
Comparison of postoperative DASH scores between HEF and IJS. *DASH*, disabilities of the arm, shoulder, and hand; *CI*, confidence interval; *MRAW*, mean raw weighted.

Subgroup analysis comparing postoperative DASH scores between HEF and IJS required the use of a random-effects model due to high overall heterogeneity (*I*^*2*^ = 98%). There was a statistically significant difference between the 2 groups (*χ*^*2*^ = 12, df = 1, *P* < .001) (Fig. 2).

### Mayo Elbow Performance Index

A total of 12 studies, including 286 patients treated with HEF,^5^^,^^15^^,^^24^^,^^26^^,^^30^^,^^33^^,^^36^^,^^40^^,^^42^^,^^46^^,^^53^^,^^55^ and 4 studies, including 76 patients treated with IJS,^9^^,^^10^^,^^44^^,^^45^ reported mean postoperative MEPI scores. Heterogeneity analysis for the HEF group yielded *I*^*2*^ = 91%, necessitating the use of a random-effects model. The pooled mean postoperative MEPI score for HEF was 86 (95% CI: 80-91). Similarly, heterogeneity analysis for the IJS group demonstrated *I*^*2*^ = 88%, prompting the use of a random-effects model, with a pooled mean postoperative MEPI score of 85 (95% CI: 77-92).

**Figure 3:**
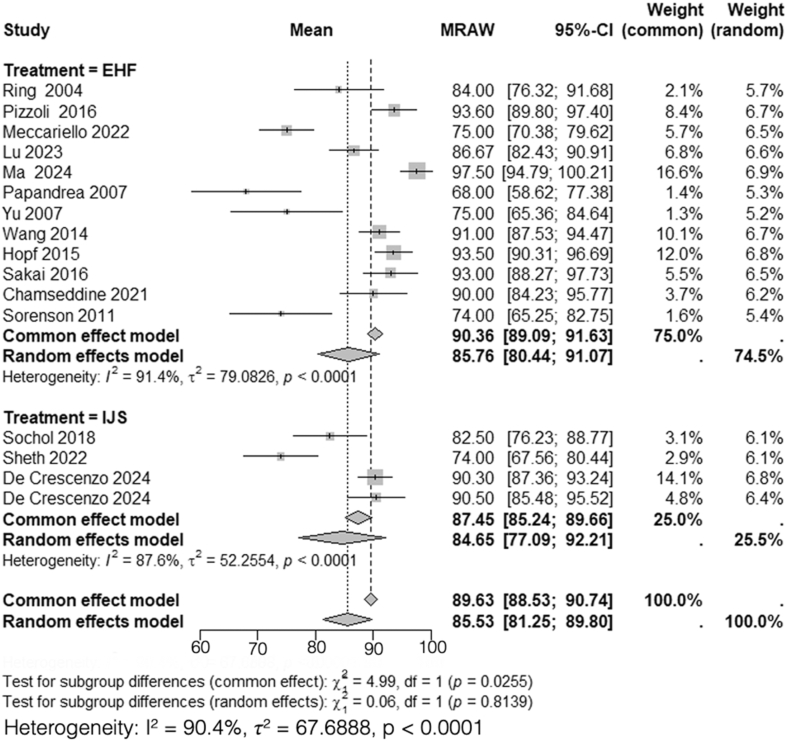
Comparison of postoperative MEPI scores between HEF and IJS. *MEPI*, mayo elbow performance index; *CI*, confidence interval; *MRAW*, mean raw weighted.

Subgroup analysis comparing postoperative MEPI scores between HEF and IJS required the use of a random-effects model due to the high heterogeneity (*I*^*2*^ = 90%). The comparison revealed no statistically significant difference between the 2 groups (*χ*^*2*^ = 0.06, df = 1, *P* = .81) (Fig. 3).

### Visual analog scale for pain

A total of 3 studies, including 72 patients treated with HEF,^24^^,^^27^^,^^36^ and 2 studies, including 26 patients treated with IJS,^9^^,^^10^ reported mean postoperative VAS scores. Heterogeneity analysis for the HEF group yielded *I*^*2*^ = 98%, necessitating the use of a random-effects model. The pooled mean postoperative VAS score for HEF was 1.8 (95% CI: 0.43-4.04). Conversely, heterogeneity analysis for the IJS group demonstrated *I*^*2*^ = 0%, and thus, a common-effects model was used. The pooled mean postoperative VAS score for IJS was 1.2 (95% CI: 1.05-1.4).

**Figure 4:**
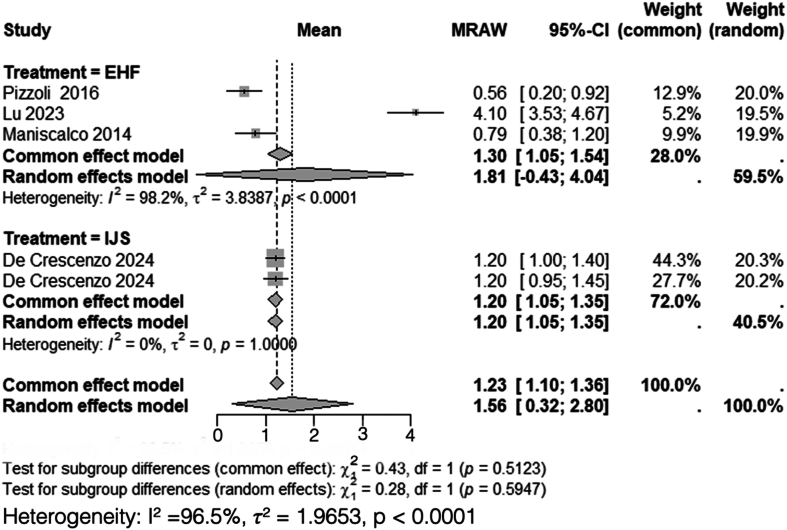
Comparison of postoperative VAS scores between HEF and IJS. *VAS*, visual analog scale; *CI*, confidence interval; *MRAW*, mean raw weighted.

Subgroup analysis comparing postoperative VAS scores between HEF and IJS required the use of a random-effects model due to high overall heterogeneity (*I*^*2*^ = 97%). The comparison revealed no statistically significant difference between the 2 groups (*χ*^*2*^ = 0.28, df = 1, *P* = .59) (Fig. 4).

### Postoperative arc of motion

A total of 17 studies, including 360 patients treated with HEF,^2^^,^^4^^,^^5^^,^^15^^,^^24^^,^^26^^,^^30^^,^^33^^,^^36^^,^^37^^,^^40^^,^^42^^,^^46^^,^^48^^,^^53^^,^^55^^,^^56^ and 7 studies, including 179 patients treated with IJS,^32^^,^^35^^,^^43^, ^44^, ^45^ reported on the mean postoperative arc of motion. Heterogeneity analysis for the HEF group yielded *I*^*2*^ = 97%, necessitating the use of a random-effects model. The pooled mean postoperative arc of motion for HEF was 115° (95% CI: 106°-124°). Similarly, heterogeneity analysis for the IJS group demonstrated I^2^ = 87%, prompting the use of a random-effects model, with a pooled mean postoperative arc of motion of 108° (95% CI: 99°-117°).

**Figure 5:**
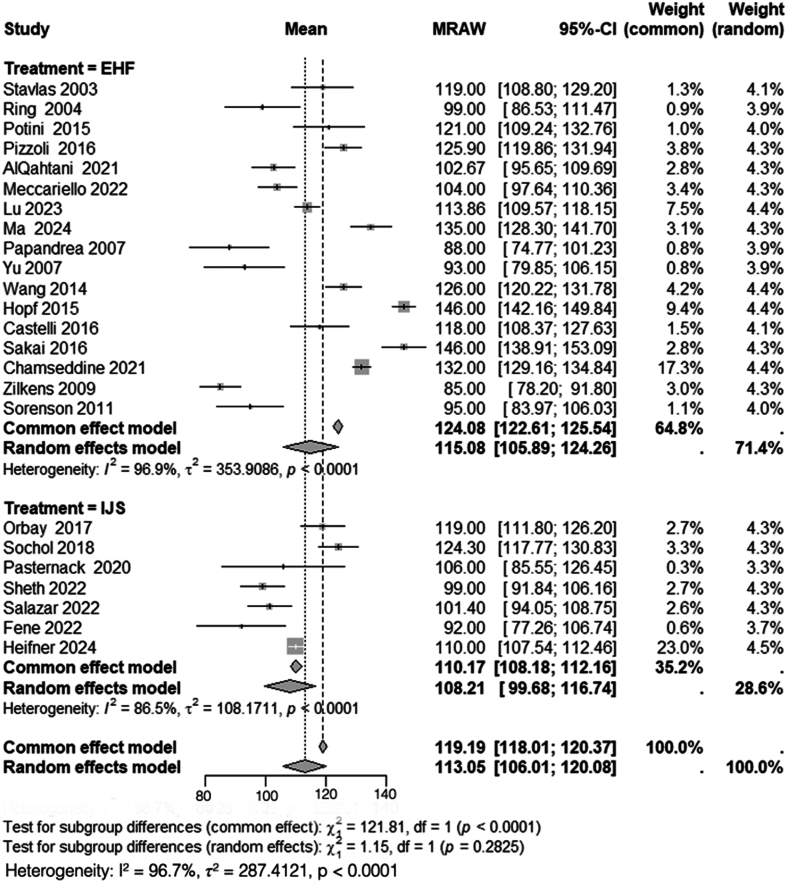
Comparison of postoperative arc of motion between HEF and IJS. *CI*, confidence interval; *MRAW*, mean raw weighted.

Subgroup analysis comparing the mean postoperative arc of motion between HEF and IJS required the use of a random-effects model due to the high heterogeneity (I^2^ = 97%). The comparison revealed no statistically significant difference between the 2 groups (*χ*^*2*^ = 1.1, df = 1, *P* = .28) (Fig. 5).

### Postoperative forearm rotation

A total of 16 studies, including 344 patients treated with HEF,^5^^,^^15^^,^^24^^,^^26^^,^^27^^,^^30^^,^^33^^,^^36^^,^^37^^,^^40^^,^^42^^,^^46^^,^^48^^,^^53^^,^^55^^,^^56^ and 4 studies, including 81 patients treated with IJS,^11^^,^^32^^,^^35^^,^^44^ reported mean postoperative forearm rotation. Heterogeneity analysis for the HEF group yielded *I*^*2*^ = 99%, necessitating the use of a random-effects model. The pooled mean postoperative forearm rotation for HEF was 136° (95% CI: 121°-151°). Similarly, heterogeneity analysis for the IJS group demonstrated *I*^*2*^ = 91%, prompting the use of a random-effects model, with a pooled mean postoperative forearm rotation of 132° (95% CI: 108°-156°).

**Figure 6:**
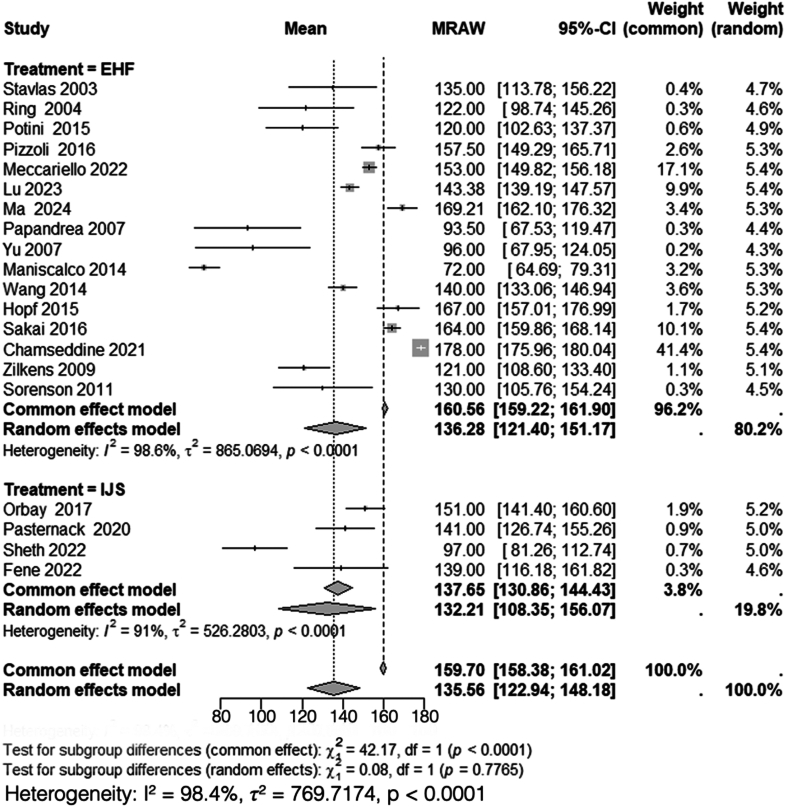
Comparison of postoperative forearm rotation between HEF and IJS. *CI*, confidence interval; *MRAW*, mean raw weighted.

Subgroup analysis comparing the mean postoperative forearm rotation between HEF and IJS required the use of a random-effects model due to the high heterogeneity (*I*^*2*^ = 98%). The comparison revealed no statistically significant difference between the 2 groups (*χ*^*2*^ = 0.08, df = 1, *P* = .78) (Fig. 6).

### Heterotopic ossification

A total of 15 studies, including 300 patients treated with HEF,^2^^,^^4^^,^^17^^,^^22^^,^^24^, ^25^, ^26^^,^^30^^,^^33^^,^^36^^,^^40^, ^41^, ^42^^,^^46^^,^^55^ and 7 studies, including 117 patients treated with IJS,^9^, ^10^, ^11^^,^^23^^,^^34^^,^^35^^,^^44^ reported on HO rates. Heterogeneity analysis for the HEF group yielded *I*^*2*^ = 71%, necessitating the use of a random-effects model. The pooled proportion of patients developing HO after HEF was 0.22 (95% CI: 0.13-0.35). Conversely, heterogeneity analysis for the IJS group demonstrated *I*^*2*^ = 38%, prompting the use of a random-effects model, with a pooled proportion of HO of 0.25 (95% CI: 0.14-0.40).

**Figure 7:**
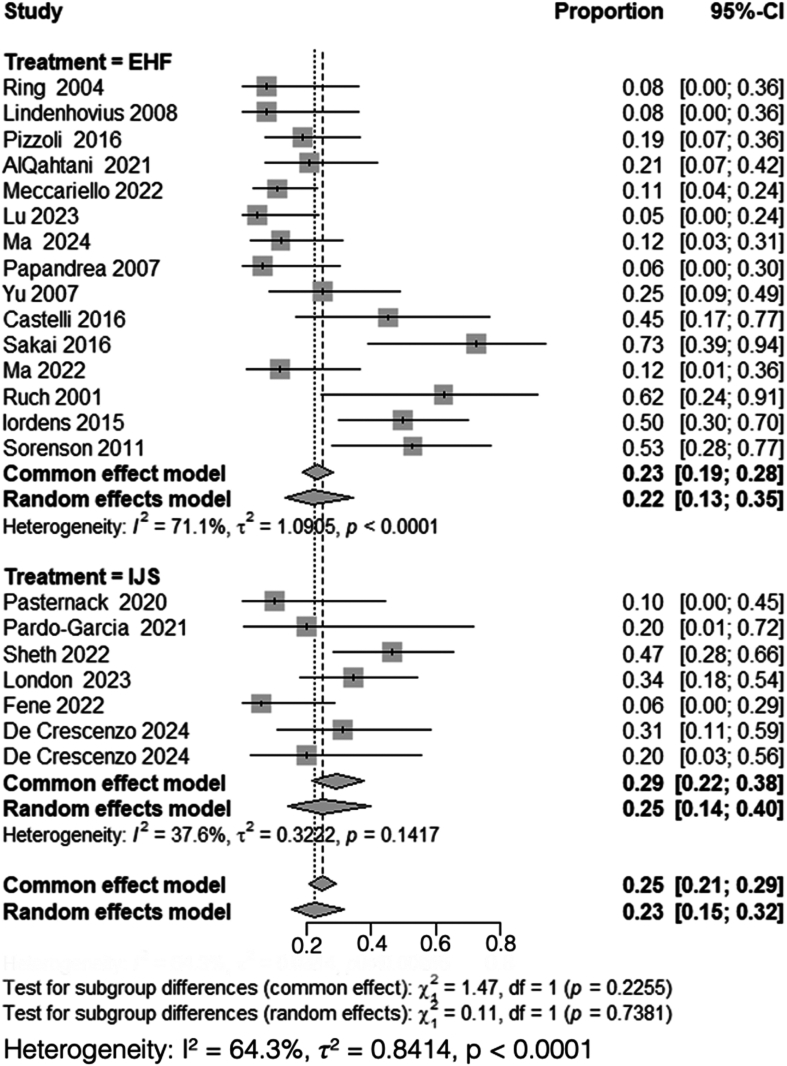
Comparison of heterotopic ossification rates between HEF and IJS. *CI*, confidence interval; *MRAW*, mean raw weighted.

Subgroup analysis comparing HO rates between HEF and IJS required the use of a random-effects model due to high overall heterogeneity (*I*^*2*^ = 64%). The comparison revealed no statistically significant difference between the 2 groups (*χ*^*2*^ = 0.11, df = 1, *P* = .74) (Fig. 7).

### Nerve injury

A total of 15 studies, including 274 patients treated with HEF,^2^^,^^4^^,^^19^^,^^22^^,^^24^^,^^25^^,^^30^^,^^33^^,^^39^^,^^41^^,^^46^^,^^48^^,^^52^, ^53^, ^54^ and 7 studies, including 122 patients treated with IJS,^9^^,^^11^^,^^23^^,^^32^^,^^35^^,^^45^^,^^54^ reported nerve injury rates. Heterogeneity analysis for the HEF group yielded *I*^*2*^ = 0%, indicating no detectable variability among studies, and thus, a common-effect model was used, with a pooled proportion of 0.11 (95% CI: 0.07-0.15). Similarly, heterogeneity analysis for the IJS group also showed *I*^*2*^ = 0%, prompting the use of a common-effect model, with a pooled proportion of nerve injuries of 0.14 (95% CI: 0.09-0.21).

**Figure 8:**
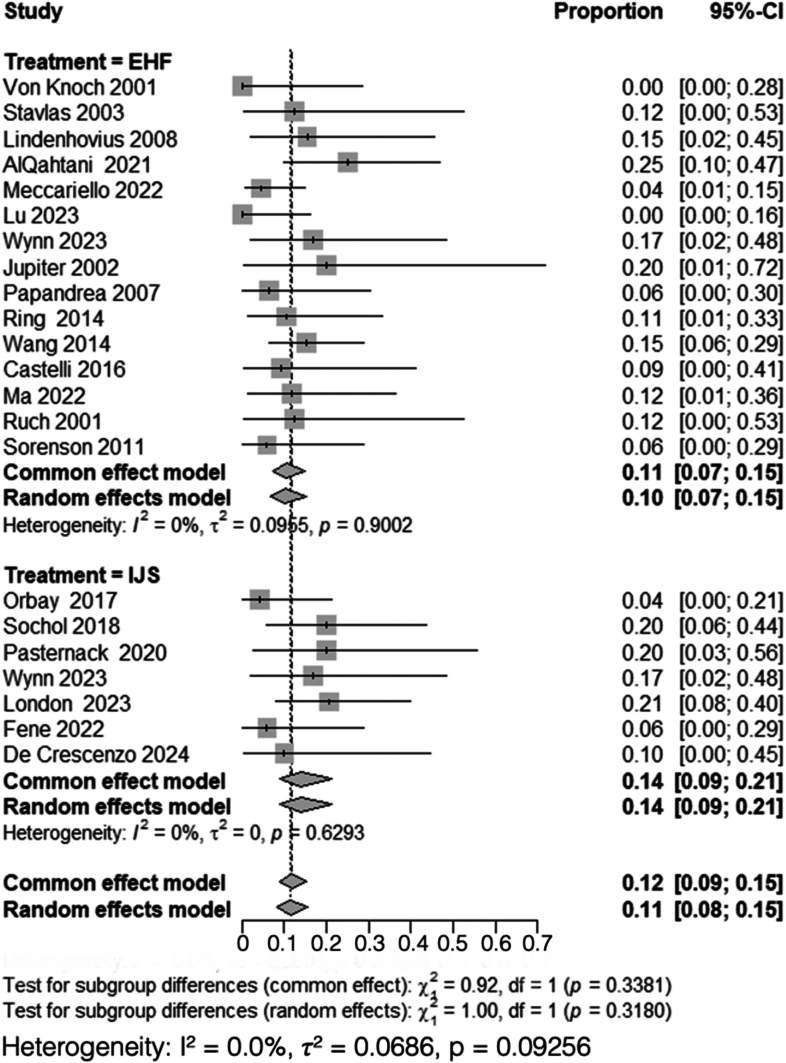
Comparison of nerve injury rates between HEF and IJS. *MRAW*, mean raw weighted.

Subgroup analysis comparing nerve injury rates between HEF and IJS required the use of a common-effect model due to overall low heterogeneity (*I*^*2*^ = 0%). The comparison revealed no statistically significant difference between the 2 groups (χ^2^ = 0.92, df = 1, *P* = .34) (Fig. 8).

### Publication bias

**Figure 9:**
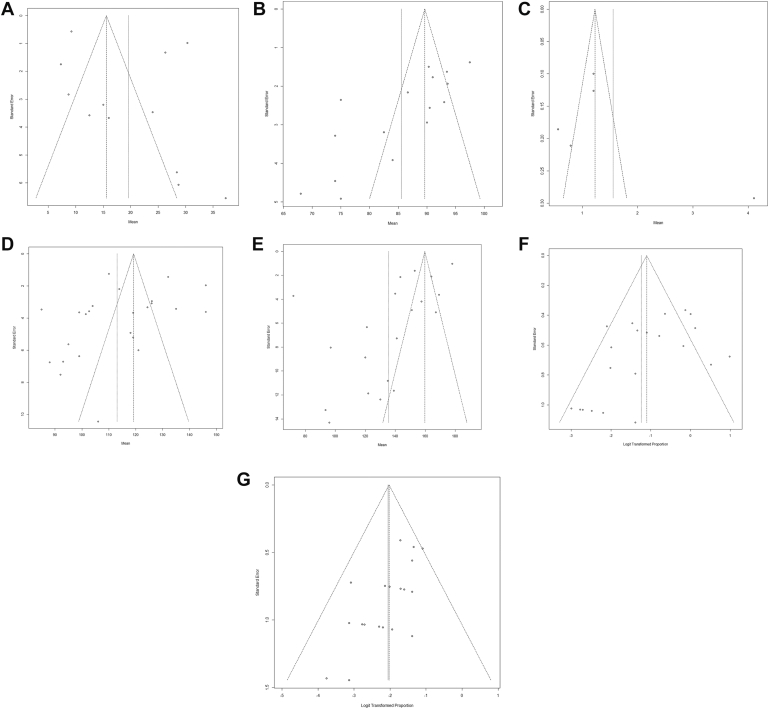
Funnel plots for publication bias: (**A**) DASH scores, (**B**) MEPI scores, (**C**) VAS scores, (**D**) Arc of motion, (**E**) Forearm rotation, (**F**) Heterotopic ossification, (**G**) Nerve injury. *DASH*, disabilities of arm, shoulder, and hand; *MEPI*, mayo elbow performance index; *VAS*, visual analog scale.

Visual inspection of the funnel plots did not reveal substantial asymmetry, suggesting minimal risk of publication bias across the analyzed outcomes (Fig. 9, *A*-*G*). The *I*^*2*^ statistic indicated that most variability among studies was due to heterogeneity rather than sampling error, with heterogeneity levels exceeding 90% for most outcome measures.

## Discussion

This systematic review and meta-analysis demonstrated a significant difference between HEF and IJS in postoperative DASH scores, with patients treated with HEF reporting better functional outcomes compared to those treated with IJS (mean DASH: 9.8 vs. 23). This 13.2-point difference exceeded the minimal clinically important difference for upper-extremity pathology, which has been reported as approximately 10.8 points by Franchignoni et al.^12^ However, no significant difference was observed in the postoperative arc of motion, forearm rotation, MEPI scores, VAS scores, HO rates, or nerve injury rates between both interventions.

Among the PROMs analyzed, MEPI and VAS scores were comparable between groups, while DASH scores were significantly better in the HEF cohort. This aligns with the findings from Wynn *et al*,^54^ who similarly reported better DASH outcomes in the HEF cohort (2.5 vs. 12), with no significant difference in MEPI scores. Taken together, these results suggest that both HEF and IJS are equally effective in restoring joint mobility and pain control, whereas HEF may provide a potential advantage in broader upper-extremity function.

Several factors may explain the discrepancy in DASH scores, including limb dominance, DASH evolution, injury severity, and rehabilitation protocols. Limb dominance is a well-recognized modifier of DASH outcomes and may have influenced the differences observed in our study. A prior study by our senior author^20^ demonstrated that DASH scores are modestly but significantly worse when the dominant limb is injured, and Bot et al^3^ similarly showed that specific DASH tasks, such as writing, are disproportionately affected when the dominant side is involved. Other investigations suggest that the effect of dominance may vary by injury type, with Christiansen et al^8^ finding no difference among patients with rotator cuff tendinopathy, while Chan et al^6^ observed worse scores when the dominant hand was involved in traumatic hand injuries. Despite this evidence, most studies in our analysis did not stratify by dominance, and reporting was inconsistent. This limitation raises the possibility that limb dominance contributed to the higher DASH scores observed in the IJS cohort.

In addition, the daily activities that can affect the DASH score have evolved, particularly those involving computer or phone use, which are no longer considered in the DASH assessment. Furthermore, the documentation of functional disabilities is now more accurate than in previous years. Given that IJS has gained popularity in recent years, these factors could partially explain why DASH scores tend to be higher in more contemporary cohorts treated with IJS compared to historical cases with HEF. Beyond patient-specific characteristics, differences in injury severity may also have influenced outcomes, as our pooled cohort ranged from simple dislocations to complex fracture-dislocations and terrible triad injuries, raising the possibility that one group included a higher proportion of severe injuries.

Finally, rehabilitation protocols following HEF and IJS differ meaningfully in duration, timing, and complexity, each influencing the patient's perceived disability. With HEF, early mobilization typically begins as soon as soft tissue conditions permit. The fixator is generally removed between 3 and 8 weeks postoperatively through a minor outpatient procedure under local anesthesia, with some patients requiring gentle manipulation at the time of removal to regain motion.^29^ In contrast, IJS protocols often involve structured therapist-supervised motion, with device removal at 6-8 weeks. Removal of internal stabilizers, however, requires a more invasive open surgery under general or regional anesthesia, given their deep placement near critical structures within the elbow joint.^25^ Although IJS eliminates the external hardware, the timing of mobilization and the burden of secondary surgery may affect the trajectory of functional recovery and ultimately PROM. These distinctions, combined with differences in injury burden and device familiarity, may underlie the observed advantage of HEF in postoperative DASH scores.

The lack of significant difference in postoperative ROM in our study aligns with the findings of Wynn et al,^54^ who found similar postoperative ROM outcomes between both HEF and IJS. However, both Wynn et al and our study did not differentiate between the variability in time from initial injury to surgical intervention. While some of the studies in our analysis stratified outcomes based on acute and chronic etiologies, the data were insufficient enough for an analysis comparing the chronicity of instability and postoperative ROM. Prior studies have demonstrated that the application of HEF in acute injuries yields a greater increase in postoperative ROM compared to its use in chronic cases,^41^^,^^46^ highlighting the potential influence of timing on treatment efficacy.

A key limitation in assessing ROM recovery is the absence of preoperative ROM values across most studies. Particularly in acute trauma settings, patients often undergo urgent operative stabilization before formal baseline assessments are recorded. Without preintervention measurements, postoperative values must be interpreted in isolation, limiting our ability to discern whether one modality leads to superior motion recovery from baseline. As a result, while our data confirm that both devices achieve acceptable and comparable postoperative arcs of motion and forearm rotation, averaging 115° and 136° for HEF, and 108° and 132° for IJS, respectively, the absence of preoperative ROM data prevents determination of which device yields greater improvement from baseline. In addition, it is important to note that loss of rotation may often result from associated injuries such as radial head fractures or malreduction rather than the stabilizing device itself. However, reporting these values provides a more complete functional profile of patients and maintains consistency with prior literature.

When comparing surgical complications between HEF and IJS, our analysis found no significant difference in the incidence of HO (22% vs. 25%) or nerve injury (11% vs. 14%). These findings suggest that both devices carry a comparable surgical risk at the time of initial implantation.

Historically, HEF has been associated with complication rates as high as 67%, primarily due to pin-site infections, nerve injuries, and hardware-related fractures.^7^^,^^39^ Recognizing these challenges, Orbay et al^31^ introduced the IJS as an implantable alternative designed to minimize these external device-related complications. The IJS is designed to stabilize the joint while permitting early motion, but with a reduced lever arm, making accurate reproduction of the axis of ulnohumeral rotation more predictable.^10^ This approach eliminates the pin-site complications commonly seen with HEF.

While our study focused solely on surgical complications, not device-related issues, our pooled analysis showed a slightly higher rate of HO and nerve injury in the IJS group. This disparity may be attributed to several factors, including smaller sample sizes, shorter follow-up periods, the relative novelty of the internal joint stabilizer technique, and the additional burden of a secondary invasive surgical procedure for device removal.^32^ In addition, a subset of patients receiving IJS had undergone prior operations requiring adjuvant stabilization, which may have predisposed them to increased complication risk.

Importantly, our findings expand upon the work of Heifner et al,^14^ who systematically compared device-related complications between IJS and HEF and observed higher rates of pin-site infections, construct failure, and recurrent instability with HEF, though these differences did not reach statistical significance.^12^ Their study highlights that the complication burden of HEF is predominantly driven by the presence and mechanics of the external hardware rather than risks inherent to the surgical implantation itself. Our findings reinforce this distinction, as we observed comparable rates of HO and nerve injury between HEF and IJS, suggesting that both devices carry a similar risk of surgical complications.

Our study is not without limitations. The predominance of retrospective data introduces inherent selection bias and limits control over confounding variables. Inconsistent reporting of a preoperative ROM precluded direct comparisons of recovery trajectories, restricting the interpretation of postoperative function to absolute values rather than true improvement. Similarly, variations in injury chronicity, surgical technique, and follow-up duration across studies likely introduced heterogeneity into our pooled estimates. Notably, rehabilitation protocols were not standardized, and the influence of therapist-directed vs. patient-guided recovery remains poorly characterized. Complication reporting may also be underpowered due to smaller sample sizes and limited longitudinal surveillance in our IJS cohort. To address these gaps, future prospective, multicenter studies with harmonized inclusion criteria, stratification by injury chronicity and dominance, and standardized rehabilitation regimens are essential. Incorporating pre- and postoperative functional scores and long-term complication surveillance will further clarify the relative advantages and limitations of HEF and IJS in managing complex elbow instability.

## Conclusion

HEF and IJS showed comparable rates of postoperative ROM, MEPI, VAS, HO, and nerve injury. However, DASH scores were 13.2 points lower in the HEF group, exceeding the minimal clinically important difference of 10.8 and indicating a clinically meaningful functional advantage of the upper extremity. This difference may be influenced by the less invasive nature of hardware removal with HEF compared to IJS. These findings should be interpreted with caution, given the overall lower level of evidence and heterogeneity across studies. Future prospective investigations with standardized rehabilitation protocols, longer follow-up, and stratification by injury chronicity, limb dominance, and preoperative motion are needed to better define optimal indications for each technique.

## Disclaimers

Funding: No funding was disclosed by the authors.

Conflicts of interest: The authors, their immediate family, or any research foundation with which they are affiliated did not receive any financial payments or other benefits from any commercialentity related to the subject of this article.

## Declaration of generative AI and AI-assisted technologies in the writing process

During the preparation of this work, the authors used ChatGPT 4.0 to improve the readability and language of select sentences originally written by the authors. After using this tool/service, the authors reviewed and edited the content as needed and take full responsibility for the content of the publication.
