# Supplementary material for: External hinged fixation vs. internal joint stabilization for elbow instability: a systematic review and meta-analysis of functional outcomes and surgical complications
Source: JSES Rev Rep Tech. 2025 Dec 11;6(2):100638. doi: 10.1016/j.xrrt.2025.100638 (PMC12876796; doi:10.1016/j.xrrt.2025.100638)
Supplement: Supplementary Table S1 [file mmc1.docx]

**Supplementary Table 1. Quality Assessment of the Cohort Studies Using the Newcastle-Ottawa Scale**

| Author, Year | Selection (Max 4★) | Comparability (Max 2★) | Exposure/Outcome (Max 3★) | Total Score (Max 9) | Quality Rating |
| --- | --- | --- | --- | --- | --- |
| Ruch, 2001^40^ | ★★★ | ★ | ★★ | 6 | Moderate |
| Von Knoch, 2001^51^ | ★★★ |  | ★★★ | 6 | Moderate |
| Jupiter, 2002^18^ | ★★★ |  | ★★★ | 6 | Moderate |
| Stavlas, 2003^46^ | ★★★ |  | ★★★ | 6 | Moderate |
| Ring, 2004^39^ | ★★★ |  | ★★★ | 6 | Moderate |
| Papandrea, 2007^32^ | ★★★ |  | ★★★ | 6 | Moderate |
| Yu, 2007^54^ | ★★★ |  | ★★★ | 6 | Moderate |
| Lindenhovius, 2008^21^ | ★★★★ | ★ | ★★★ | 8 | High |
| Zilkens, 2009^55^ | ★★★ |  | ★★★ | 6 | Moderate |
| Sorenson, 2011^48^ | ★★★ |  | ★★★ | 6 | Moderate |
| Maniscalco, 2014^26^ | ★★★ |  | ★★★ | 6 | Moderate |
| Ring, 2014^38^ | ★★★★ | ★ | ★★★ | 8 | High |
| Wang, 2014^52^ | ★★★ |  | ★★★ | 6 | Moderate |
| Hopf, 2015^14^ | ★★★ | ★ | ★★★ | 7 | High |
| Iordens, 2015^16^ | ★★★ | ★ | ★★★ | 7 | High |
| Potini, 2015^36^ | ★★★ |  | ★★★ | 6 | Moderate |
| Castelli, 2016^4^ | ★★★ |  | ★★★ | 6 | Moderate |
| Pizzolo, 2016^35^ | ★★★ |  | ★★★ | 6 | Moderate |
| Sakai, 2016^41^ | ★★★ |  | ★★★ | 6 | Moderate |
| Orbay, 2017^31^ | ★★★ |  | ★★★ | 6 | Moderate |
| Sochol, 2018^44^ | ★★★ |  | ★★★ | 6 | Moderate |
| Pasternack, 2020^34^ | ★★★ |  | ★★★ | 6 | Moderate |
| AlQahtani, 2021^2^ | ★★★★ | ★★ | ★★★ | 9 | High |
| Chamseddine, 2021^5^ | ★★★ |  | ★★★ | 6 | Moderate |
| Pardo-Garcia, 2021^33^ | ★★ |  | ★★★ | 5 | Moderate |
| Fene, 2022^11^ | ★★★ |  | ★★★ | 6 | Moderate |
| Ma, 2022^24^ | ★★★ | ★ | ★★★ | 7 | High |
| Meccariello, 2022^29^ | ★★★ |  | ★★★ | 6 | Moderate |
| Salazar, 2022^42^ | ★★★ |  | ★★★ | 6 | Moderate |
| Sheth, 2022^43^ | ★★★ | ★ | ★★★ | 7 | High |
| London, 2024^22^ | ★★★ |  | ★★★ | 6 | Moderate |
| Lu, 2023^23^ | ★★★★ | ★ | ★★★ | 8 | High |
| Uddin, 2023^49^ | ★★★ |  | ★★★ | 6 | Moderate |
| Wynn, 2023^53^ | ★★★★ | ★ | ★★★ | 8 | High |
| De Crescenzo, 2024^9^ | ★★★ | ★ | ★★★ | 7 | High |
| De Crescenzo, 2024^10^ | ★★★ | ★ | ★★★ | 7 | High |
| Heifner, 2024^13^ | ★★★ |  | ★★★ | 6 | Moderate |
| Ma, 2024^25^ | ★★★ | ★ | ★★★ | 7 | High |
