# Supplementary material for: External hinged fixation vs. internal joint stabilization for elbow instability: a systematic review and meta-analysis of functional outcomes and surgical complications
Source: JSES Rev Rep Tech. 2025 Dec 11;6(2):100638. doi: 10.1016/j.xrrt.2025.100638 (PMC12876796; doi:10.1016/j.xrrt.2025.100638)
Supplement: Supplementary Table S2 [file mmc2.docx]

**Supplementary Table 2. Quality Assessment of the RCT using the Revised Cochrane Risk-of-bias tool for randomized trials (RoB 2)**

| **Author, Year** | **Randomization Bias** | **Deviation from Intended Interventions** | **Missing Outcome Data** | **Measurement Bias** | **Selective Reporting Bias** | **Overall Risk of Bias** |
| --- | --- | --- | --- | --- | --- | --- |
| Lu, 2023^23^ | Low – Digital randomization platform used, 1:1:1 allocation, well-described concealment | Low – Postoperative care protocol was consistent; adherence monitored; blinding of outcome assessors stated | Low – All 65 patients followed; none lost to follow up | Low – Outcome assessors were blinded; validated scores (MEPS, DASH, VAS) used | Low – All outcomes prespecified and fully reported; trial registry number provided | Low risk of bias |
