# Supplementary material for: External hinged fixation vs. internal joint stabilization for elbow instability: a systematic review and meta-analysis of functional outcomes and surgical complications
Source: JSES Rev Rep Tech. 2025 Dec 11;6(2):100638. doi: 10.1016/j.xrrt.2025.100638 (PMC12876796; doi:10.1016/j.xrrt.2025.100638)
Supplement: Supplementary Figure S1 [file mmc3.docx]

**Supplementary Figure 1: Search strategy for Pubmed and Google Scholar**

**Pubmed**

internal stabilizer AND elbow instability OR internal joint stabilization AND elbow instability OR internal stabilization AND elbow instability OR external fixation OR fixator AND elbow instability OR hinged fixation OR fixator AND elbow instability OR static fixation OR fixator AND elbow instability

**Google Scholar**

("internal stabilizer" OR "internal joint stabilizer" OR "external fixation" OR "hinged fixator" OR "elbow fixator") AND "elbow instability" NOT (humerus OR shoulder OR "case report" OR "systematic review" OR "meta-analysis")
